# Monocular depth sensing using metalens

**DOI:** 10.1515/nanoph-2023-0088

**Published:** 2023-03-28

**Authors:** Fan Yang, Hung-I Lin, Peng Chen, Juejun Hu, Tian Gu

**Affiliations:** Department of Materials Science and Engineering, Massachusetts Institute of Technology, Cambridge, MA 02139, USA; Department of Physics, Peking University, Beijing 100871, China

**Keywords:** depth sensing, double-helix, metalens, metasurface

## Abstract

3-D depth sensing is essential for many applications ranging from consumer electronics to robotics. Passive depth sensing techniques based on a double-helix (DH) point-spread-function (PSF) feature high depth estimation precision, minimal power consumption, and reduced system complexity compared to active sensing methods. Here, we propose and experimentally implemented a polarization-multiplexed DH metalens designed using an autonomous direct search algorithm, which utilizes two contra-rotating DH PSFs encoded in orthogonal polarization states to enable monocular depth perception. Using a reconstruction algorithm that we developed, concurrent depth calculation and scene reconstruction with minimum distortion and high resolution in all three dimensions were demonstrated.

## 1 Introduction

Conventional optical imaging systems map 3-D scene to a flat image plane at the cost of losing depth information. The missing knowledge of object distances, however, is crucial to a variety of applications spanning autonomous driving, object recognition, gesture control, virtual/augmented reality, etc. Multiple active depth sensing mechanisms have been utilized to retrieve 3-D information, such as time-of-flight and structured light [1–9]. However, these sensing techniques require active illumination and modulation components, which add to system complexity, cost and power consumption. Passive stereo cameras infer object distance information by comparing different images captured at different viewpoints of the scene. However, they are limited by the well-known trade-off between system size and depth resolution.

An alternative route is depth-from-defocus (DFD), which applies computational imaging techniques to infer the depth from defocus blur of a classical lens [10–17]. However, defocus cue is often ambiguous and requires complementary information such as pictorial depth cues to determine the depth. They also have low depth estimation accuracy since a defocused point-spread-function (PSF) of a classical lens varies slowly along the optical axis. In addition, the DFD method further suffers from limited depth range and degraded lateral resolution. To solve these issues, PSF engineering has been explored to enhance depth discrimination capability. This approach employs custom tailored phase masks to define PSF of the system, and depth information can be encoded into the captured image directly. PSFs designed for depth estimation include astigmatic PSF [18], biplane PSF [19], tetrapod-like PSF [20–22], etc. Among them, double-helix (DH) PSF [23–28] generates two rotating foci, where the rotation angle determines the object depth. This method streamlines image data post-processing given its shape simplicity. To produce the phase mask for DH PSF generation, the classical approach involves a spatial light modulator (SLM) placed in the Fourier plane of a 4f system, which however creates alignment challenges and significantly increases footprint of the entire system.

Metasurfaces provide a compact and cost-effective alternative to the 4f system. They are composed of sub-wavelength nanostructures that furnishes on-demand control of the outgoing wavefront [29–36]. Compared to diffractive optical elements (DOE) [37–39], metasurfaces offer much higher spatial resolution in phase mask definition, which contributes to higher efficiency, elimination of high-order diffraction, reduced aberration of PSFs, and increased degrees of freedom for wavefront modulation. A monocular DH metasurface was experimentally realized by Jin et al. [40]. The image captured by the DH metasurface is the convolution of the scene with the DH PSF, and thus the depth information can only be estimated with prior knowledge of the original object. Colburn et al. coupled a DH metasurface with an extended depth-of-focus (EDOF) metasurface in a binocular setting to resolve this ambiguity [41]. Multiplexing presents a way to combine the two metasurfaces into one aperture to realize monocular depth estimation (MDE) [42]. Along this line, MDE was recently demonstrated with a decoupled pair of conjugate single-helix PSFs [43].

In this paper, we demonstrate a polarization-multiplexed DH metasurface design for MDE using a single metasurface. Two DH PSFs with opposite rotating directions are each encoded with a linear polarization. Importantly, the focal point rotation angles of the two PSFs always add up to 90°, a feature that allows computationally efficient and unambiguous reconstruction of both the depth and image. As one specific example, we experimentally implemented the design at 635 nm wavelength within the depth range of 45–212 mm and rotation angles of up to 80°. This concept is generically applicable to other wavelengths and depth ranges.

## 2 Design principle of metalens with contra-rotating DH PSFs

The DH phase mask is constructed based on superposition of Laguerre–Gaussian modes [44–46], it modifies the wavefront emitted from a point-source to generates two foci on the image plane, where orientation of the line connecting the foci depends on the point source distance. Rotation rate and depth range are determined by the choice of the Laguerre–Gaussian mode set. In previous implementations, a block-iterative weighted projection algorithm was utilized to optimize the DH phase mask and suppress the focal spot sidelobes, thereby improving depth estimation accuracy [41, 46, 47]. However, the optimization procedure is a time-consuming empirical process which requires constant human intervention. Here, we use a direct search (DS) algorithm [48–52] to optimize the DH phase mask. The figure-of-merit (FOM) for the DS is defined in Eqs. (1)–(3). It comprises the sum of the lower intensities between the two foci over a discrete set of sampling rotation angles, minus variance of the intensities between different angles. Here, *I*_1_(*ϕ*) and *I*_2_(*ϕ*) are the intensities of the two foci of a certain rotation angle *ϕ*, *c*(*ϕ*) compensates for illumination intensity decrease of the point source at increasing distance, *z*(*ϕ*) denotes the distance of the point source, *N* represents the total number of rotation angles sampled, *S* ^2^{*I*(*ϕ*)} calculates the variance of the intensities among different angles, and *κ* = 1 is a weighting factor. Equation (4) gives the relationship between rotation angle and distance using the same notations as in Ref. [41], with *V*_1_ and *ω*_0_ being free parameters to control the rotation rate and depth range.

(1) $$FOM=\frac{1}{N}\overset{N}{\underset{i=1}{\sum }}I\left(\phi_{i}\right)-\kappa \cdot S^{2}\left\{I\left(\phi_{i}\right)\right\}$$

(2) $$I\left(\phi \right)=\min\left\{I_{1}\left(\phi \right),I_{2}\left(\phi \right)\right\}\cdot c\left(\phi \right)$$

(3) $$c\left(\phi \right)=z{\left(\phi \right)}^{2}$$

(4) $$z=\frac{\pi \omega_{0}^{2}}{\lambda }\tan\left(\frac{\phi_{0}-\phi }{V_{1}}\right)$$

With a DH phase mask, an image formed through the metalens is the convolution of the DH PSF and the object. Without prior knowledge of the object, it is not possible to extract the true object information from a single-shot image. We eliminate this ambiguity by multiplexing two DH phase masks with opposite rotation directions into a single metalens. The two phase masks share an identical layout albeit with their *x* and *y* coordinates swapped, and therefore only one phase mask design is required. After acquiring two images corresponding to the two polarization states, a series of deconvolution operation is performed on them assuming DH PSFs of varying rotation angles. Since the two images capture the same object, their deconvolved outcome should be identical provided that a correct rotation angle is used. Therefore, by identifying the deconvolved image pair with maximum similarity, the rotation angle and hence object depth can be unambiguously determined. More details of the depth retrieval algorithm are discussed in Section 4. Further improvement of the design is possible leveraging the rise of end-to-end optimization framework in recent years, which provides an alternative approach to optimize both the meta-optical frontend and the reconstruction algorithm [53–55].

**Figure 1:**
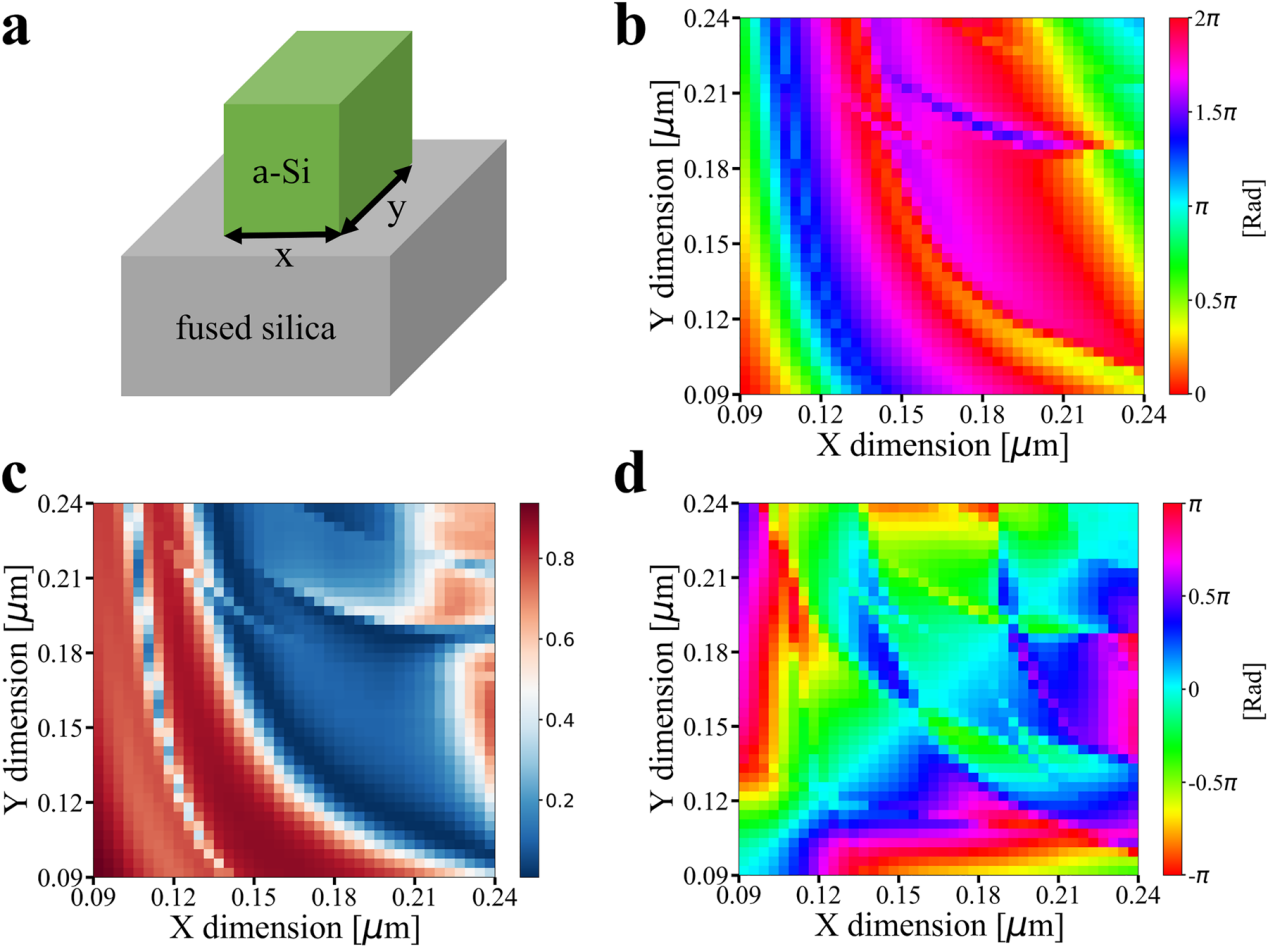
Polarization-multiplexed meta-atom design. (a) Illustration of the meta-atom structure. (b) Phase delay and (c) transmittance of the meta-atoms with *x*-polarized incident light. (d) Phase delay difference between the two polarization states.

An approach similar to that in Ref. [56] is employed to design polarization-multiplexed meta-atoms. The meta-atom structure is schematically depicted in Figure 1(a), comprising a 450 nm thick rectangular amorphous silicon nano-post sitting on a fused silica substrate. The geometries of the nano-posts are designed according to the polarization directions of the incident light. The meta-atom pitch is fixed at 300 nm. The finite-difference time-domain (FDTD) method is used to analyze the meta-atom responses. The phase delay and transmittance of the meta-atoms under *x*-polarized incident light are shown in Figure 1(b) and (c). Data pertinent to *y*-polarized light can be trivially obtained by swapping the *x* and *y* coordinates. The phase difference between two polarization states is shown in Figure 1(d). A 2-bit design [57] containing 16 meta-atom structures of different lateral dimensions is chosen to allow independent control of the metalens’ phase profiles in both polarization states. The meta-atom dimensions are listed in the Appendix.

## 3 Metalens fabrication and PSF characterization

As a proof of concept, we designed a metalens with 1 mm aperture size and 5 mm focal length. Parameters in Eq. (4) are taken as *ω*_0_ = 125, *ϕ*_0_ = 180° and *V*_1_ = 2, identical to those in Ref. [41]. The DS optimization was performed with *N* = 41 in Eq. (1), i.e. with 41 evenly spaced discrete rotation angles at a step size of 2°. The designed metalens has a rotation angle up to 80°, corresponding to a depth sensing range of 45–212 mm.

**Figure 2:**
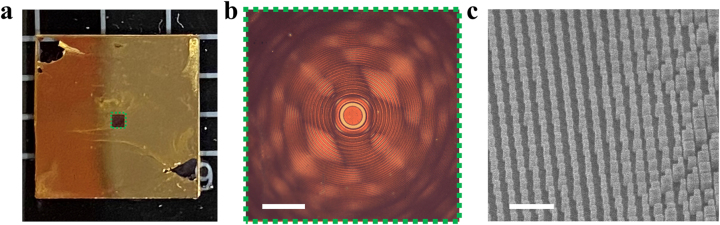
Images of the fabricated metalens. (a) Fabricated metalens on the silica substrate with metal mask. (b) Optical microscope image of the metalens. (Scale bar: 200 μm). (c) Scanning electron microscope image of the metalens. (Scale bar: 1 μm).

The so-designed metalens was fabricated through electron-beam lithography followed by reactive-ion etching. Figure 2 presents optical microscope and scanning electron microscope (SEM) images of the fabricated metalens, showing excellent uniformity and pattern fidelity.

**Figure 3:**
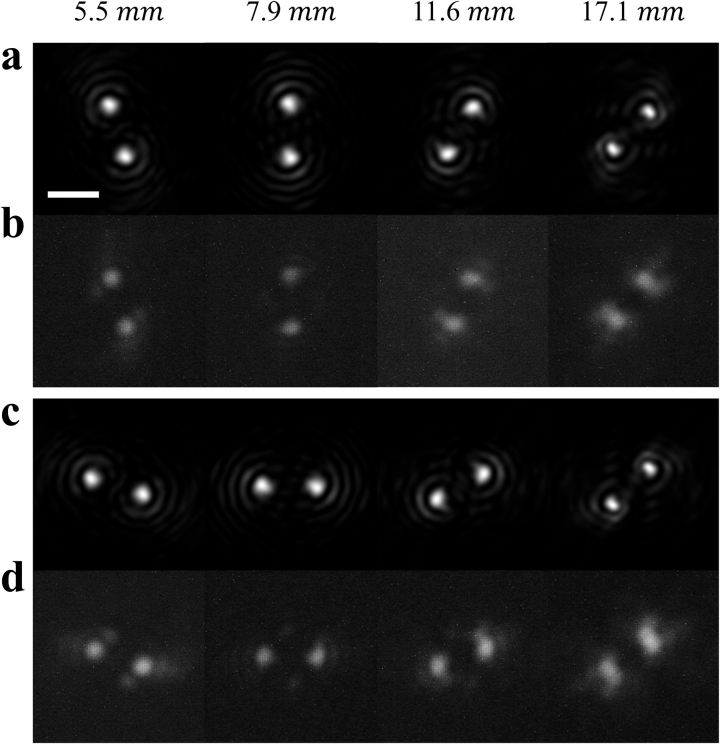
Metalens PSF. (a) Simulation and (b) experimental measurement of PSFs for different source distances in the *x* polarization state. (c) Simulation and (d) experimental measurement of PSFs in the *y* polarization state. (scale bar: 20 μm).

The PSF of the fabricated metalens was first characterized. Figure 3 compares the simulated and measured PSFs with different rotation angles. In the simulation, Kirchhoff diffraction integral [58] is used to transform the near-field wavefront after exiting the metasurface to the intensity distribution on the image plane. During the PSF measurement, a monochrome micro-LED display (Jade Bird Display 5000DPI AMuLED Panel) is placed in front of the metalens at different distances, and a 40 μm diameter circular spot is displayed to emulate a point object. A telescope assembly is placed between the DH metalens and an image sensor (Arducam MT9J001) with a calibrated magnification of 5. A polarizer is mounted in front of the sensor to control the polarization state of the incident light. As seen from Figure 3, excellent agreement is obtained between our design and experiment throughout the entire depth range.

## 4 Concurrent imaging and depth mapping demonstration

**Figure 4:**
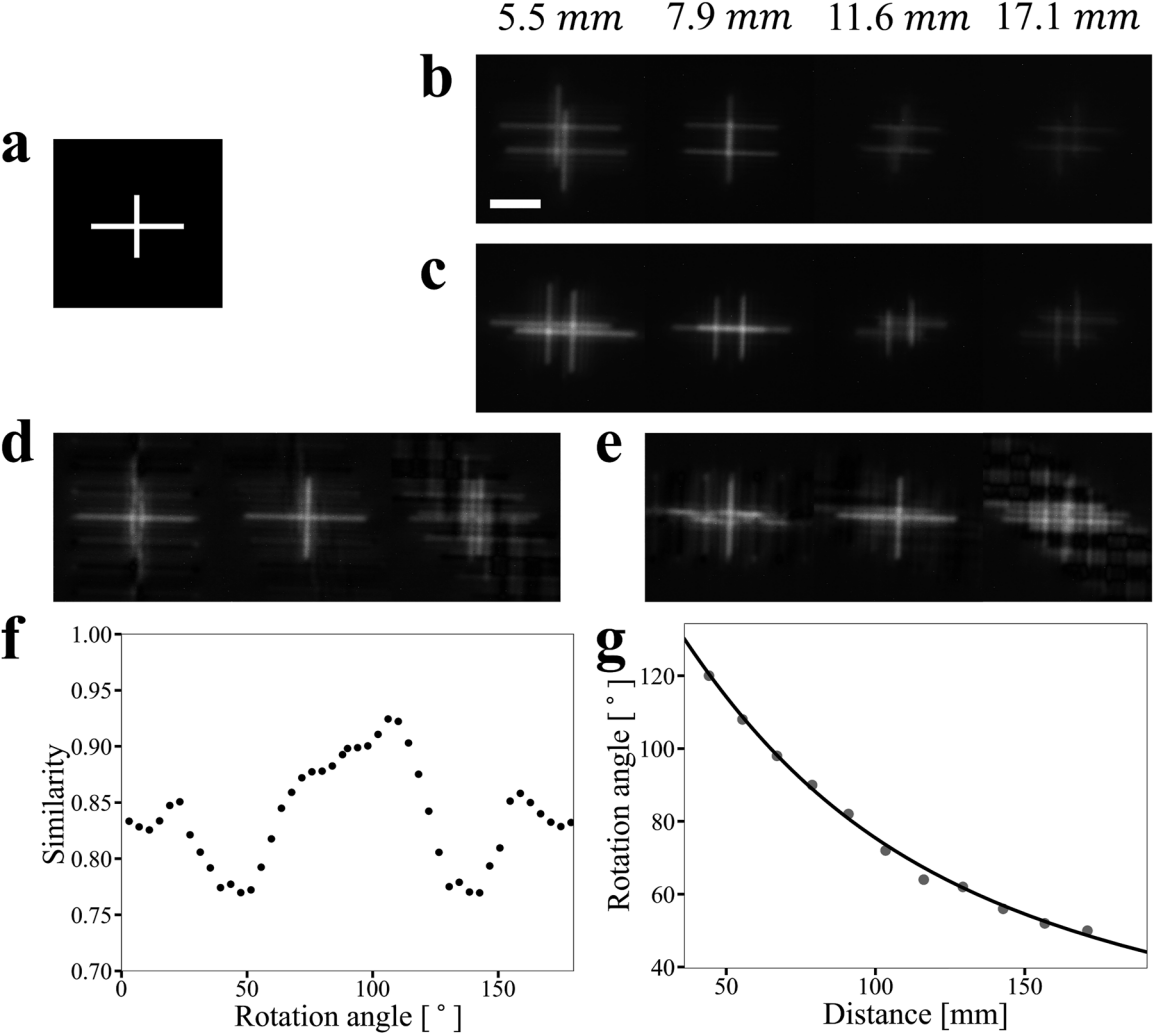
Experimental demonstration of image deconvolution to enable concurrent depth mapping and scene reconstruction. (a) A ‘+’ pattern on the micro-LED display emulates an object. (b, c) Images in the (b) *x*-polarization state and (c) *y*-polarization state with different object distances. (d) Deconvolved images of the object at 5.5 cm distance using DH PSF rotation angles of 90°, 110°, and 130° (left to right), respectively. (e) Deconvolved images of the object at 5.5 cm distance with DH PSF rotation angles of 0°, −20°, and −40° (left to right), respectively. (f) Similarity of image pairs deconvolved using different DH PSF rotation angles. The maxmium point corresponds to the correct rotation angle. (g) Object depth estimation based on analytical expression (solid line) and experimental measurement (red dots). (Scale bar: 40 μm).

The experimental setup consists of the micro-LED display projecting a ‘+’-shaped pattern shown in Figure 4(a) and placed at varying distances. The images captured by the DH metalens are shown in Figure 4(b) and (c) for the two polarization states, respectively. To extract depth information and reconstruct the scene, these images are deconvolved using a set of PSF pairs with different rotation angles. Since the two phase masks corresponding to orthogonal polarizations are linked via a reflection transformation, the sum of rotation angles of every PSF pair must equal 90°. We use Wiener deconvolution given in Eq. (5), where *H* is the Fourier transform of the image formed by the lens, *G* gives the Fourier transform of DH PSF (i.e., a cosine function), *F* denotes the Fourier transform of the deconvolved image, and *SNR* = 0.1 is the signal-to-noise ratio, which is an intrinsic parameter of the image sensor.

(5) $$F=\frac{H\cdot G}{G^{2}+SNR}$$

Three pairs of deconvolved images in two polarization states are shown in Figure 4(d) and (e), each assuming a different rotation angle. We then computed the image correlation map between the image pair using Eq. (6), where ‘corr’ stands for the image correlation map, and *h*_1_ and *h*_2_ represent the deconvolved image pair. We further define the similarity parameter as the maximum value within the image correlation map, and Figure 4(f) plots the parameter as a function of the rotation angle. Since the pair of images depict the same object, the similarity curve should reach maximum when the rotation angles of the DH PSFs used in the deconvolution are correct. The object depth can then be inferred according to the correct rotation angles.

(6) $$corr\left(x,y\right)=\int h_{1}\left(x^{\prime },y^{\prime }\right)\cdot h_{2}\left(x^{\prime }-x,y^{\prime }-y\right)dx^{\prime }dy^{\prime }$$

The protocol described above was applied to depth estimation of objects placed at different distances, and the measured depth values are shown as red dots in Figure 4(g). The analytical expression of Eq. (4) that our lens design is based on is also plotted as a solid line, showing excellent agreement.

**Figure 5:**
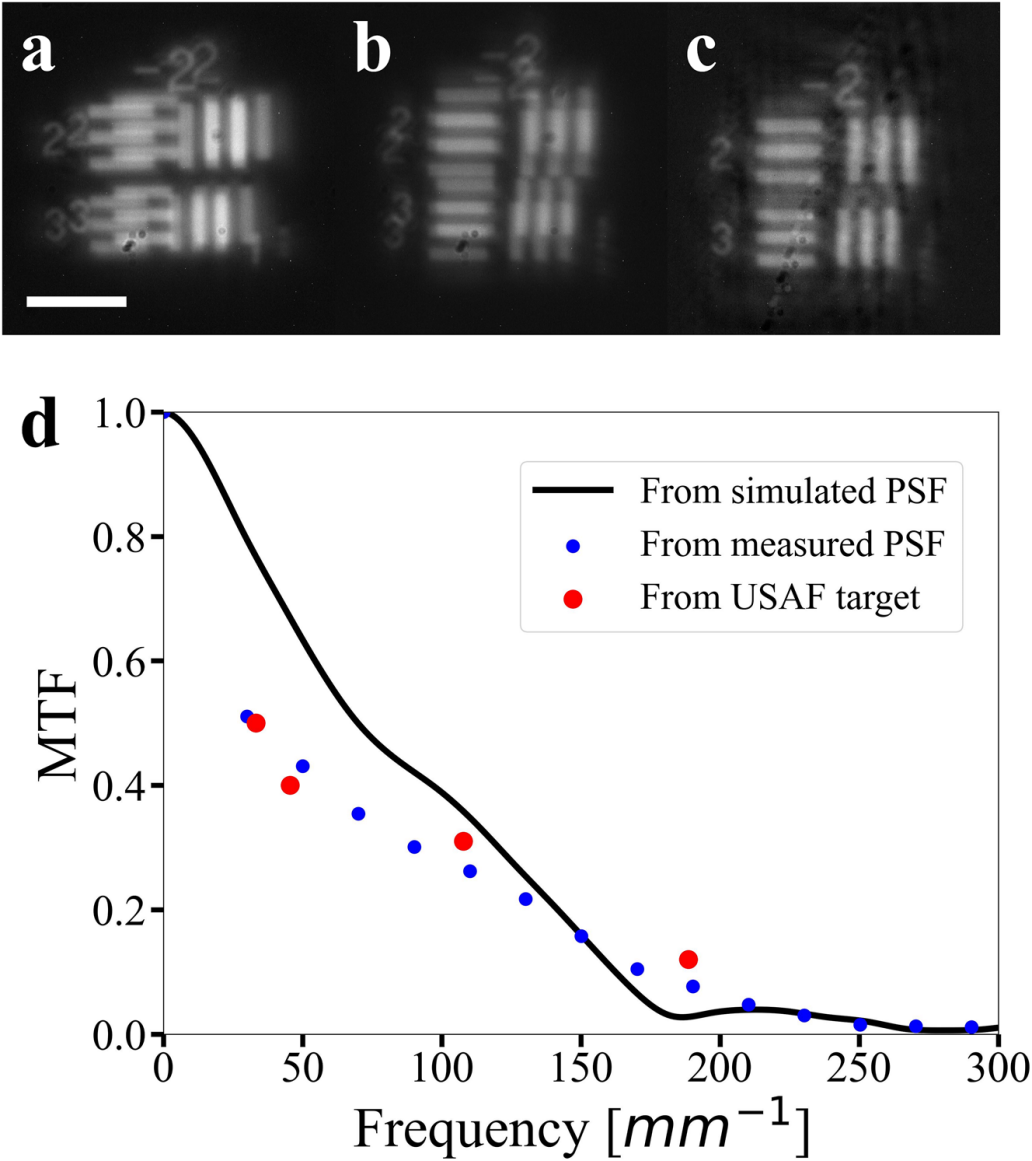
Imaging of the USAF target for evaluating the lateral resolution. Experimentally captured images in the (a) *x*-polarization and (b) *y*-polarization states. (c) Reconstructed image of the USAF resolution target pattern. (d) Measured MTF of the metalens at an object distance of 5.5 mm. The red dots correspond to MTF measured from the USAF target and the blue dots are MTF calculated from experimentally measured PSFs via Fourier transform. The solid line gives MTF inferred from PSFs simulated by diffraction integral. (Scale bar: 80 μm).

To further characterize the lateral spatial resolution of the DH metalens, we replaced the ‘+’ pattern on the micro-LED display with a standard USAF resolution chart. As an example, the captured images under *x* and *y* polarized light are shown in Figure 5(a) and (b) for an object distance of 5.5 mm. The same deconvolution algorithm was performed to reconstruct the scene shown in Figure 5(c). The modulation transfer function (MTF) at different spatial frequencies was obtained from the image contrast of the reconstructed resolution chart. In addition to the direct MTF measurement, we also evaluated the MTF from Fourier transform of the measured DH PSFs. Both sets of results are plotted in Figure 5(d) with excellent agreement, which assures accuracy of our reconstruction algorithm.

**Figure 6:**
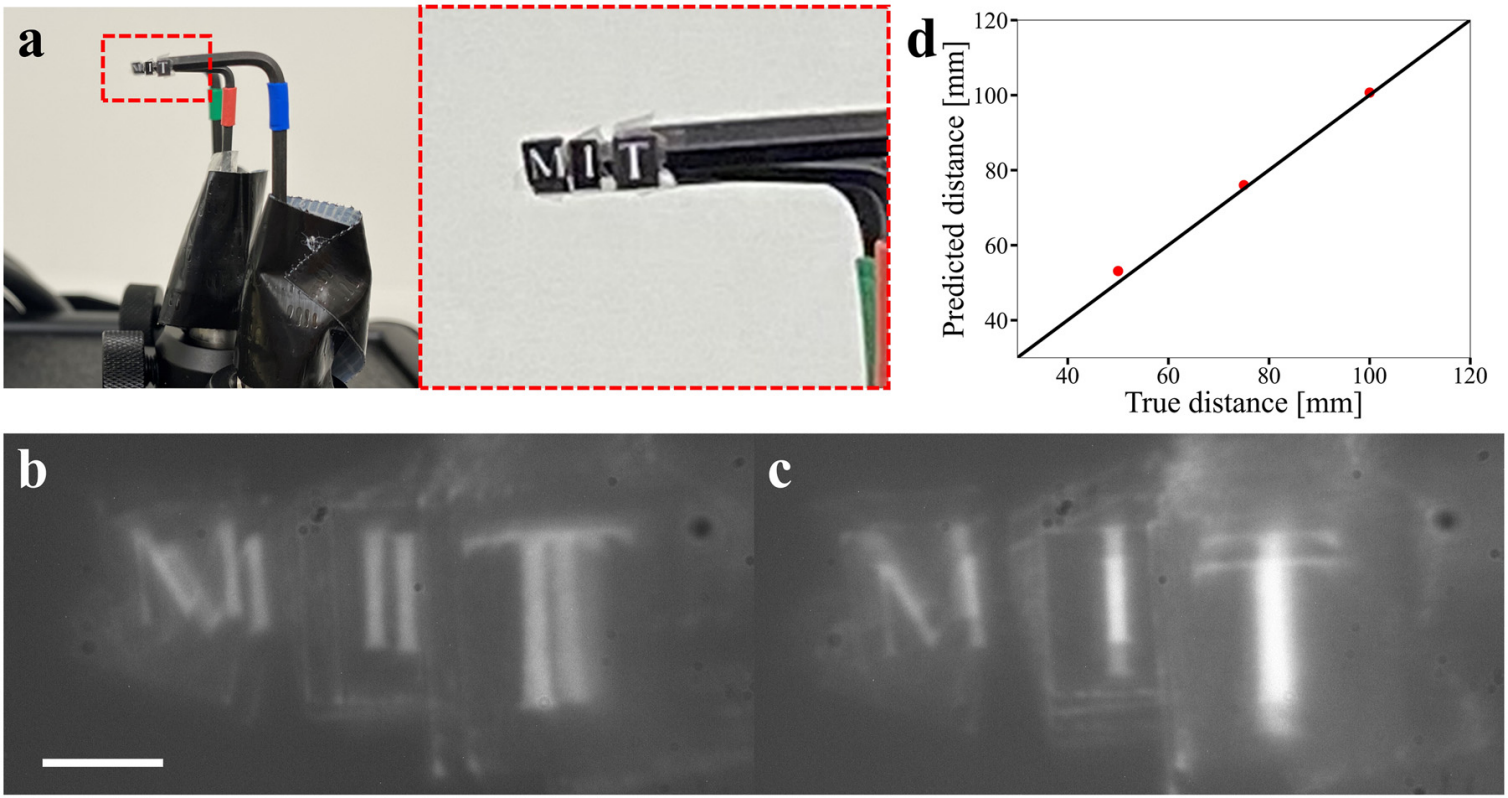
Experimental demonstration of depth sensing. (a) Photos of printed letters of ‘M’, ‘I’, and ‘T’, each placed at a different distance. (b, c) Captured images in (b) the *x*-polarization state and (c) the *y*-polarization state. (d) Inferred object distances (red dots) compared to the ground truth (solid line). (Scale bar: 80 μm).

Lastly, real world imaging was demonstrated using letters of ‘M’, ‘I’, and ‘T’ printed on card boards. The letters were displaced at different distances as shown in Figure 6(a). An LED light source with a center wavelength of 625 nm and 20 nm full-width-at-half-maximum (FWHM) spectral bandwidth was used to illuminate the scene, and a filter with 635 nm center wavelength and 10 nm FWHM bandwidth was placed in front of the image sensor to reject out-of-band light. The images recorded in the two polarization states are shown in Figure 6(b) and (c). The aforementioned algorithm was implemented to infer the depth of the objects, with the caveat that oblique incidence onto the metalens (which leads to additional phase delay) was accounted for and corrected following procedures outlined in Ref. [41]. The extracted depths are shown in Figure 6(d), which agrees well with the ground truth. The average error of depth estimation is 2.7%.

## 5 Conclusions

We demonstrated a monocular metalens design capable of perform both depth sensing and scene reconstruction concurrently. The design leverage polarization-multiplexing to encode two phase masks, each generating an optimized DH PSF. Our design ensures that rotation angles of the two contra-rotating PSFs are always complementary, which enables unambiguous depth perception without prior knowledge of the scene. Compared to other depth sensing methods, our MDE approach features a single-aperture, compact footprint, high depth and lateral resolution, and passive operation. These advantages foresee vast potential applications of our technology in areas such as microscopy, medical imaging, virtue/augmented reality, automotive/robotic sensing and beyond.

## Appendix A: Polarization-multiplexed meta-atom design

The meta-atom dimensions and their phase/transmittance responses are listed in Table 1.

**Table 1:** Polarization-multiplexed meta-atoms.

Meta-atom index	1	2	3	4	5	6	7	8
*X* dimension [nm]	136	159	159	148	90	98	90	90
*Y* dimension [nm]	140	90	98	109	159	98	117	132
*X*-pol phase [°]	−3	−17	−4	−10	125	87	83	98
*Y*-pol phase [°]	5	125	183	273	−17	87	180	274
*X*-pol transmittance	0.87	0.89	0.89	0.89	0.81	0.88	0.89	0.86
*Y*-pol transmittance	0.88	0.81	0.76	0.76	0.89	0.88	0.74	0.78
**Meta-atom index**	**9**	**10**	**11**	**12**	**13**	**14**	**15**	**16**
*X* dimension [nm]	98	117	228	105	109	132	121	117
*Y* dimension [nm]	159	90	228	121	148	90	105	117
*X*-pol phase [°]	183	180	185	186	273	274	261	264
*Y*-pol phase [°]	−4	83	185	261	−10	98	186	264
*X*-pol transmittance	0.76	0.74	0.70	0.74	0.76	0.78	0.77	0.77
*Y*-pol transmittance	0.89	0.89	0.70	0.77	0.89	0.86	0.74	0.77
